# Evaluation of Self-Inflicted versus Non-Self-Inflicted Gunshot Wounds and Associated Injuries Involving the Hand and Upper Extremity

**DOI:** 10.3390/healthcare12050564

**Published:** 2024-02-29

**Authors:** Tommy Pan, Brianne M. Giuffrida, Amol H. Trivedi, Dom Contestabile, Praveer S. Vyas, Boyle C. Cheng, Daniel T. Altman, Steven M. Regal

**Affiliations:** 1 Allegheny Health Network Orthopaedic Institute, Pittsburgh, PA 15212, USA; tpan12345@gmail.com (T.P.); bmg332@drexel.edu (B.M.G.); trivedi.amol@ahn.org (A.H.T.); dom.contestabile@ahn.org (D.C.); praveer.vyas@ahn.org (P.S.V.); daniel.altman@ahn.org (D.T.A.); steven.regal@ahn.org (S.M.R.); 2 Drexel University College of Medicine, Drexel University, University City Campus, Philadelphia, PA 19104, USA

**Keywords:** gunshot wound, hand, upper extremity, complications, trauma, management, surgery

## Abstract

Orthopedic costs associated with gunshot wounds (GSWs) totaled approximately USD 510 million from 2005 to 2014. Previous studies have identified differences in injuries associated with self-inflicted (SI) GSWs; however, there remains a gap in understanding injury patterns. This study aims to expand upon the current literature and shed light on injury patterns and outcomes associated with SI vs. non-self-inflicted (NSI) GSWs. This is a retrospective cohort study of upper extremity GSWs from January 2012 to December 2022. Data were analyzed using the two-sample *t*-test, Pearson’s chi-squared test, and Fisher’s exact test. SI GSWs tended to be high-velocity GSWs and occurred more often in distal locations compared to NSI GSWs (*p* = 0.0014 and *p* < 0.0001, respectively). SI GSWs were associated with higher Gustilo–Anderson (GA) and Tscherne classifications (*p* < 0.0001 and *p* = 0.0048, respectively) and with a greater frequency of neurovascular damage (*p* = 0.0048). There was no difference in fracture rate or need for operative intervention between the groups. GA and Tscherne classifications were associated with the need for and type of surgery (*p* < 0.0001), with a higher classification being associated with more intricate operative intervention; however, GSW velocity was not associated with operative need (*p* = 0.42). Our findings demonstrate that velocity, wound grading systems, and other factors are associated with the manner in which GSWs to the upper extremity are inflicted and may thus have potential for use in the prediction of injury patterns and planning of trauma management and surgical intervention.

## 1. Introduction

More than half of all estimated households in the United States report having a firearm [1]. A retrospective review of the Medicare national patient record database from 2005 to 2014 identified approximately 9700 gunshot wound (GSW) injuries involving orthopedic care, with about 2200 of those requiring operative intervention. Nonfatal GSWs lead to significant expenses for the healthcare system, as the orthopedic costs associated with GSW alone totaled about USD 510 million [2].

Approximately 17% of all nonfatal gunshots and 70% of unintentional gunshot wounds involve the upper extremity [3]. Gunshot fractures often do not follow injury patterns observed in blunt injury scenarios, leading to complex and challenging stabilization. GSW injuries to the upper extremity can greatly impact patient function, fine motor skills, and the ability to return to work [4]. Up to 65% of patients that required operative intervention were unable to work due to chronic pain [4]. 

Previous studies have begun to identify the differences in high-velocity injuries associated with self-inflicted GSW injuries; however, there is still a need to further understand injury patterns [5]. Due to the proximity of neurovascular structures within the hand and distal upper extremity, hand and upper extremity surgeons need to have an understanding of the mechanism and patterns to help guide optimal treatment decisions. The goal of this retrospective cohort study is to expand upon the existing literature to better understand the differences in self-inflicted (SI) vs. non-self-inflicted (NSI) GSW injuries with regard to injury patterns and outcomes among different patients.

## 2. Materials and Methods

Institutional review board approval was obtained for this retrospective cohort study. From January 2012 to December 2022, all consecutive cases of a GSW to the upper extremity were identified using the relevant Current Procedural Terminology codes in a query of the institution’s Epic electronic health record system. Patient characteristics included: age, gender, ethnicity, body mass index (BMI; kg/m^2^), history of hypertension (HTN), diabetes mellitus (DM), or peripheral vascular disease (PVD), smoking history, and type of occupation. Additional data regarding the GSW were collected including type of gun used, type of GSW (self-inflicted versus non-self-inflicted), injury locations, and multiple wounds (>1 bullet wound). Open fracture was classified according to the Gustilo–Anderson (GA) and Tscherne classifications. Nerve injury type was categorized according to Seddon Criteria (neurapraxia, axonotmesis, and neurotmesis). The primary outcomes measured included location of injury, duration until antibiotics given (hours), fracture pattern, initial management of injury, definitive surgical fixation, and associated nerve or vascular injury. Secondary objectives included duration of follow-up, duration until return to work, number of trips to the operating room, number of specialty consults and complications, and length of hospital stay (days). Continuous data were analyzed using a two-sample *t*-test (for pooled variance or unequal variances) and are reported as mean ± standard deviation (SD). Categorical data were analyzed using Pearson’s chi-squared test and Fisher’s exact test and are reported as count (%). *p* < 0.05 was considered statistically significant. The specific group sample sizes *n* for variables for which data were missing are provided in the tables. 

## 3. Results

### 3.1. Epidemiology

From January 2012 to December 2022, 295 patients were identified to have a GSW to the upper extremity. After individual review, 22 patients were found to be miscoded for a GSW or did not have adequate data available and were not included. A final sample of *N* = 273 patients was used. Of the 273 GSW cases, 100 were SI and 173 were NSI. Demographics were compared between these two groups. 

Patients who experienced a GSW to the upper extremity were mostly male. There was no significant difference in the distribution of gender between SI and NSI groups. Patients with SI GSWs (47.7 ± 19.5 years) were significantly older than those with NSI GSWs (32.2 ± 12.6 years; *p* < 0.0001). Patients with SI GSWs also had a significantly higher BMI (*p* = 0.0045); however, this difference may not be clinically significant due to the effect size and relatively large variances (Table 1). The SI group was predominantly (88%) white whereas the NSI group was predominantly (75.7%) non-white (*p* < 0.0001). Of the occupations examined (manual, skilled, or retired/unemployed), the SI group comprised mostly manual (54%) and retired/unemployed (37%) workers. The NSI group had a significantly higher proportion of retired/unemployed patients (63%), followed by manual workers (31.2%; *p* = 0.0002). There was no significant difference in the history of HTN, DM, PVD, or smoking between the SI and NSI groups. Patient characteristics are summarized in Table 1. 

Among the different demographic characteristics examined (age, sex, BMI, and ethnicity), only ethnicity was associated with GSW velocity (*p* = 0.0024); non-white patients were more likely to sustain a low-velocity GSW than white patients.

### 3.2. Gunshot Wound Characteristics

The most common gun used in a SI GSW was a handgun (91%) followed by a shotgun (6%) and a rifle (3%), whereas NSI GSWs were almost exclusively (98.8%) caused by a handgun (*p* = 0.0036). The gunshot velocity was determined based on the type of gun used if not explicitly stated in the patient chart. Low velocity was attributed to a handgun and high velocity was attributed to a shotgun or rifle. The SI group had a higher number of high-velocity GSWs (15%) compared to the NSI group (4%; *p* = 0.0014).

The five most common GSW locations in the SI group were the hand (85%), forearm (6%), shoulder (3%), elbow (2%), and humerus (2%). The five most common GSW locations in the NSI group were the hand (33%), forearm (22%), humerus (19.1%), shoulder (16.2%), and elbow (4.1%). SI GSWs were mostly localized to the hand, and NSI GSWs were more distributed across the upper extremity. Similarly, a higher proportion of SI GSWs were localized more distally than NSI GSWs. The NSI group had more patients with multiple injury locations (3.5%) and more cases of multiple wounds (33.7%) when compared to the SI group (8% multiple wounds and 1% multiple injury locations) (*p* < 0.0001). Upon arrival at the emergency department, more patients from the SI group received antibiotics (95%) compared to the NSI group (83.8%; *p* = 0.0063). There was no significant difference in rate of fracture or need for operating room (OR) intervention between the groups. The results are summarized in Table 2. 

### 3.3. Injury Characteristics

SI GSWs were associated with a higher incidence of nerve injury (45%) compared to NSI GSWs (30.1%; *p* = 0.013). The most common nerve injury type in the SI group was neuropraxia (27%) followed by axonotmesis (14%) and neurotmesis (4%). In the NSI group, axonotmesis was the most common injury type (16.2%) followed by neuropraxia (13.9%), and no neurotmesis was observed. The types of nerve injury differed significantly between the groups (*p* = 0.0035). Among patients with nerve injury, there was a higher number of digital nerve injuries in the SI group (66.7%) than in the NSI group (16.1%; *p* < 0.0001). The digital nerve was the most common nerve injured and the ulnar nerve was the second most common nerve injured (20%) in the SI group. In the NSI group, the ulnar nerve was the most commonly injured (33.9%) and the radian/PIN nerve was the second most common (19.4%) nerve injured. The only significant difference was the higher number of digital nerve injuries in the SI group (*p* < 0.0001). 

SI GSWs were also associated with a higher incidence of vascular injury (11%) than NSI GSWs (3.5%; *p* = 0.013). The digital (72.7%), ulnar (18.2%), and radial (9.1%) arteries were the most common vessels injured in the SI group, and the brachial (50%), ulnar (16.7%), and digital (16.7%) arteries were the most common vessels injured in the NSI group. The arteries injured differed significantly between the groups (*p* = 0.017). SI GSWs were associated with a higher incidence of tendon injury (25%) than NSI GSWs (10.4%; *p* = 0.0014). There were limited injuries to muscle bodies and no significant difference in muscle injury between the groups. The results of the GSW injury characteristics are summarized in Table 3. 

### 3.4. Hypotheses Related to GSW Velocity, Wound Classification, and Surgical Intervention

We hypothesized that a higher GSW velocity would be associated with higher GA and Tscherne classification, worse neurovascular damage, and surgical intervention. Among patients requiring surgical intervention, we hypothesized that higher-velocity GSW would be associated with more intricate surgical intervention. We also predicted that higher GA and Tscherne classifications would be associated with requiring surgery and more intricate types of surgical intervention.

There were significant differences identified in both GA and Tscherne classifications between SI and NSI GSW fractures (*p* < 0.0001 and *p* = 0.0048, respectively). Most SI GSWs were classified as GA Type 2 (67%); 17% were Type 3; and the remaining 16% were Type 1. NSI GSWs were also most likely to be classified as GA Type 2 (53.8%), but this was significantly less than in SI GSWs. NSI GSWs also showed a high count of GA Type 1 (39.9%), and only 6.4% of cases were GA Type 3. Both SI and NSI groups had a Tscherne classification of I or II; however, SI GSWs had a Tscherne classification of IV as the third most common compared to NSI GSWs who had a classification of III as the third most common. The results of the GSW classifications are summarized in Table 4.

GSW velocity was associated with neurovascular damage (Fisher’s exact test, *p* = 0.0048). Low GSW velocity was associated with no neurovascular damage, followed by neuropraxia, nerve palsy, and then nerve laceration. High GSW velocity was associated with either no neurovascular damage or nerve palsy, followed by neuropraxia and then nerve laceration. 

Of the 273 included cases, 148 were non-surgical and 125 involved surgical intervention. Type of surgery was coded as follows: 0 = Irrigation and Debridement (I&D), 1 = Open Reduction Internal Fixation/Closed Reduction Percutaneous Pinning (ORIF/CRPP), 2 = External Fixation, 3 = Neurovascular (N/V) repair OR fasciotomy/Carpal Tunnel Release (CTR), 4 = Other (includes exploratory laparotomy, flap surgery, removal of hardware, and release). Regarding the surgical subgroup, it was found that there was a significant association between type of surgery and GA classification (*p* < 0.0001), as well as type of surgery and Tscherne classification (*p* = 0.0005). In the cases with a Type 1 GA classification, the most common surgical procedures were ORIF/CRPP and Other. In the cases with a Type 2 GA classification, the most common operation was ORIF/CRPP. Cases with a Type 3 GA classification had N/V repair or fasciotomy/CTR as the most common operation. The most common operation for cases with Tscherne Grade I or II injuries was ORIF. Tscherne Grade III injuries were equally likely to be treated with either ORIF or N/V repair or fasciotomy. Patients with Grade IV injuries most often underwent N/V repair or fasciotomy. GSW velocity was not associated with the need for surgical intervention (*p* = 0.39) or the type of surgery (*p* = 0.42). 

## 4. Discussion

With the large prevalence of gun ownership in United States households, nonfatal gunshot wounds have led to significant expenses for the healthcare system. In a retrospective review by Rosas et al., investigators analyzed the Medicare national patient record database from 2005 to 2014 and found that orthopedic costs accounted for over USD 510 million. Additionally, it is worth mentioning the impact of upper extremity gunshot wounds on the greater economy. These injuries often impair patient function and the ability to return to work, with 65% of patients who underwent operative intervention being unable to work due to chronic pain [6]. 

Self-inflicted GSWs have been shown to make up 81.4% of GSWs to the hand [7], yet there remain limited studies that explore patterns of injury for self-inflicted GSWs in the upper extremity. Van Handel et al. compared basic epidemiology differences between SI and NSI GSW injuries but did not evaluate the differences in injury patterns between SI and NSI groups. The goal of our study was to (1) characterize the difference between SI and NSI GSW injuries, (2) describe injury patterns, and (3) identify factors associated with different types of GSWs that could be considered in a clinical setting to improve trauma management. We hypothesized that a higher GSW velocity would result in higher GA and Tscherne classification, worse neurovascular damage, surgery in the OR, and more intricate types of surgical intervention. We also predicted that higher GA and Tscherne classifications would be associated with the need for surgery and more intricate types of surgical interventions.

The classic presentation of SI GSW injuries is an accidental discharge while cleaning the firearm, usually a handgun [7]. Previous studies found that 92.7% of all firearm injuries to the upper extremity presented with fracture, among which 92.2% had a GA classification score of at least 3A [8]. Nerve injury is a common complication of gunshot wounds to the upper extremity, with rates of nerve injury ranging from 15 to 70%. It is clear that GSW injuries can result in a large range of damage that requires different protocols that utilize more or less resources depending on the severity. This broad range in possible treatment plans makes pattern recognition of GSW severity important in medical decision making. 

The demographic results of our study found older, white males to be most likely to have self-inflicted GSWs and younger, non-white males to have non-self-inflicted GSWs. This is consistent with previous studies evaluating the epidemiology of SI versus NSI GSWs [3,5,7,9]. Further, we found that SI GSWs were most likely to occur in manual workers, while NSI GSWs were most likely to occur in retired/unemployed individuals. However, a recent study by Krzeczowski et al. examined the racial disparity and was unable to attribute it to population demographics or socioeconomic status, indicating a need to further investigate this correlation [9]. 

The most common gun used in both SI and NSI GSWs was a handgun, which is consistent with data released by the Bureau of Alcohol, Tobacco, Firearms and Explosives in 2023 that stated pistols were the most frequently traced crime gun (68%) [10]. Guns used in SI GSWs differed from those used in NSI GSWs in that there were more cases of shotgun and rifle use in the former, while a handgun was almost exclusively used in the latter population. SI GSWs are usually an accidental discharge in the context of cleaning, hunting, etc. [7], and this accounts for the difference in the type of gun used. 

In this study, 85% of SI GSWs and only 33% of NSI GSWs were to the hand. This is consistent with previous studies that have identified a greater incidence of SI GSWs to the hand [3,5,7,11]. Previous studies have examined specific injury locations within the hand and identified SI GSWs to be associated with the most distal structures (proximal phalanges) and NSI GSWs to be associated with more proximal structures (metacarpals) [3,7]. The current study examined injury locations across the entire upper extremity. SI GSWs were most likely to be within the hand, forearm, shoulder, elbow, and humerus. NSI GSWs were most likely to be within the hand, forearm, humerus, shoulder, and elbow, but compared to the SI group where injuries were focused on the hand, NSI GSWs demonstrated a more uniform injury distribution across the upper extremity. The NSI group also demonstrated a higher incidence of multiple wounds. This is consistent with previous studies and is most likely attributed to a violent context rather than an accidental discharge [3,5]. There was no difference in fracture rate between the groups with fractures occurring in 50–60% of the cases. There was no difference in the need for operative intervention between the groups, with 40–50% of each group receiving OR intervention. These results differ from our original hypothesis that the SI group would require more surgical intervention. Previous studies have stated that around 30–50% of SI GSWs require OR interventions, with the most OR interventions needed for more distal injuries [5,7,11], and up to 65% of self-inflicted GSW cases require surgical treatment and have a higher incidence of multiple OR trips [3]. 

The difference in the results from previous studies may be due to confounding variables such as the high number of multiple wounds within the NSI GSW group requiring more surgical intervention despite the lower-energy GSWs. Although there was no difference in fracture rate or surgical intervention, the SI GSW group did receive more antibiotics than the NSI GSW group. This may be due to the higher velocity associated with SI GSWs and their affinity to more distal structures. Most of the cases of NSI GSWs that did not receive antibiotics were due to a superficial laceration to the shoulder or proximal arm from a GSW graze. There still remains a lack of consensus on antibiotic prophylaxis in GSWs [12,13], indicating a need to further examine this for medical decision making. 

One variable that contributes to predicting the extent of tissue and structural damage is the velocity of the bullet, with higher-velocity GSWs being associated with greater tissue and structural damage [4,6,14]. SI GSWs were more likely to result in nerve, vascular, and tendon injury when compared to NSI GSWs. SI GSWs resulted in neuropraxia to the digital nerve and vascular damage to the digital artery, whereas NSI GSWs more commonly lead to nerve palsy of the ulnar nerve. The affiliated injuries demonstrated a similar pattern to the location of GSWs. The SI group had nerve and vascular injuries localized to the distal nerves and arteries (digital and ulnar), and the NSI group’s nerve injuries demonstrated a more uniform nerve and artery distribution across the upper extremity (ulnar, radial, median, and digital nerves). Previous studies have found that upper extremity fractures associated with GSWs predict nerve damage [13,15]; however, the current study shows that there was a higher incidence of nerve injuries associated with the SI group despite no differences in fracture rate between the groups. Therefore, there are other variables that contribute to nerve injuries, and our results indicate that gunshot velocity predicts associated nerve damage rather than fracture rate. Other studies evaluating injuries associated with GSWs found a predominance of neuropraxic events [3,15] that is consistent with the results of the SI group. 

A higher GA fracture type and Tscherne classification were seen in the SI group, with most fractures being GA Type 2 and 3 and Tscherne Class I, II, and IV. The NSI group was most likely to be GA Type 1 and 2 and Tscherne Class I, II and III. One study found that 92.2% of GSW fractures had a GA classification of at least 3A [8]; however, our results show much lower fracture severities, with only 17% of SI and 6.4% of NSI GSWs being GA Type 3. This difference could be attributed to our larger sample size and more representative population, in contrast to the predominantly rural population studied by McIlrath et al. These significant findings lend support to our hypothesis that higher-velocity GSWs, more of which are seen in SI GSWs, can predict the severity of GA type as well as Tscherne classification. 

It was found that GA and Tscherne classification are associated with the need for and type of surgical intervention; however, GSW type (SI versus NSI) is not. GA classifications of 1 and 2 were associated with ORIF/CRPP, and a classification of 3 was associated with N/V repair or fasciotomy/CTR. A Tscherne Grade of I or II was associated with ORIF, and a Grade III was equally associated with ORIF or N/V repair or fasciotomy/CTR. This is consistent with our hypothesis that higher GA and Tscherne classifications are associated with the need for surgery and more intricate operations. Previous studies have found that GSWs with a single entrance wound with no soft tissue, vascular, or bone injury can be managed without surgical intervention (local debridement, splinting, and antibiotics), and GSWs with fractures or soft tissue injuries are best managed with early ORIF reconstruction with or without bone grafting [12]. Additionally, any tendon or nerve repair as well as any need for flap coverage should be managed early for best post-operative functionality [12]. Our results add to this data by providing metrics—GA and Tscherne classification—that could be used to assist in predicting operative intervention and aid in clinical triage. 

Our data highlight the differences in injury patterns and clinical outcomes between SI and NSI GSWs and suggest potential associations between multiple factors (classification systems, velocity, type of surgical intervention, etc.). We hope these findings can assist in clinical decision making in a trauma setting to avoid suboptimal management leading to increased morbidity in patients with nonfatal GSWs. Follow-up studies should be conducted to better understand the differences between the types of injury that these factors are associated with, and the specific care required. It has been reported that within orthopedic surgery alone, nonfatal GSW cases can result in high utilization of resources and multiple surgical procedures [2]. The investigators of this epidemiological study suggest promoting physician-driven policy initiatives to decrease the overall incidence of GSWs [2]. Although we agree, we hope that our findings encourage further investigation specifically into current clinical management to identify ways to deliver efficient care and reduce healthcare costs.

This study has potential limitations. Due to the retrospective nature of this study, data were limited to what was documented at each encounter. Data such as velocity and type of gun used were subject to interpretation based on the narrative of the history of present illness (HPI) when not explicitly stated. When velocity was not explicitly stated, velocity was assumed based on the type of gun used, with low velocity being attributed to a handgun and high velocity being attributed to a shotgun or rifle. When the type of gun was not explicitly mentioned, the HPI narrative and context of the incident was used to collect data on the type of gun used. Most cases of SI GSWs stated the type of weapon used. For NSI GSW cases where the type of gun was not mentioned, the mention of a “hunting incident” in the HPI was associated with the use of a rifle, any mention of ‘pistol’ was associated with the use of a handgun, and shootings associated with crime (such as a drive by shooting, streetside shooting, or attempted homicide) were associated with a handgun. This assumption was made based on the Bureau of Alcohol, Tobacco, Firearms and Explosives identifying pistols as the most used weapon [10]. Given the lack of specific bullet velocity data and the assumptions that were made thereafter, due to the inherent difficulty in acquiring such data, our findings may not reflect the true nature of specific GSWs. Another limitation to this study is that for some of the statistical analysis, a single value had to be chosen in instances where there was more than one value. This occurred when there was more than one location of GSW in patients with multiple wounds, more than one nerve that was impacted/damaged, and more than one surgical procedure performed in a trip to the operating room. In these instances, the more serious injury location, more seriously damaged nerve, or most relevant procedure was chosen as the single value. This may disrupt the associations gathered from the data. As a result, there may be an underlying association not explored between the duality of GSW location, nerve damage, or surgical procedures with other variables of interest. 

## 5. Conclusions

This study examined the differences in management and outcomes between SI (high-velocity) and NSI (low-velocity) GSWs to expand upon the current literature on upper extremity gunshot trauma. We found that SI GSWs were localized more distally, associated with more neurovascular damage, and had higher GA and Tscherne classifications when compared to NSI GSWs. Additionally, NSI GSWs were found to be more uniformly distributed across the upper extremity, and despite our SI GSW findings, neither group showed a difference in fracture rate or surgical intervention when compared to one another. When assessing GA and Tscherne classification, a higher grade was associated with the need for operative intervention and more intricate procedures. In contrast, GSW velocity was not associated with more intricate procedures nor was it associated with the need for surgical intervention in the OR.

Our findings suggest that velocity, wound grading systems, and other factors may have potential use in the prediction of injury patterns and planning of trauma management and surgical intervention. Further studies should be conducted to evaluate these and other factors as accurate predictors of patient outcomes, and to investigate operative outcomes such as estimated blood loss, complications, and recovery time. We hope that this information can be used to help guide clinical decision making in patients who suffer from gunshot trauma to the upper extremity.

## Figures and Tables

**Table 1:** Patient Characteristics (*N* = 273).

	Type of Gunshot Wound		
	SI (*n* = 100)	NSI (*n* = 173)	*p*
Age (years)	47.7 ± 19.5	32.2 ± 12.6	<0.0001
Sex			0.35
Male	91 (91.0%)	151 (87.3%)	
Female	9 (9.0%)	22 (12.7%)	
Ethnicity			<0.0001
White	88 (88.0%)	42 (24.3%)	
Non-White	12 (12.0%)	131 (75.7%)	
BMI (kg/m^2^)	30.7 ± 7.7	28.1 ± 7.1	0.0045
Hypertension			0.0042
No	72 (72.0%)	149 (86.1%)	
Yes	28 (28.0%)	24 (13.9%)	
Diabetes Mellitus			0.69
No	93 (93.0%)	163 (94.2%)	
Yes	7 (7.0%)	10 (5.8%)	
Peripheral Vascular Disease			0.99
No	100 (100.0%)	173 (100.0%)	
Yes	0 (0.0%)	0 (0.0%)	
Smoking			0.28
No	49 (49.0%)	73 (42.2%)	
Yes	51 (51.0%)	100 (57.8%)	
Occupation			0.0002
Manual	54 (54.0%)	54 (31.2%)	
Skilled	9 (9.0%)	10 (5.8%)	
Retired/Unemployed	37 (37.0%)	109 (63.0%)	

**Table 2:** Gunshot Wound Characteristics (*N* = 273). The effective sample size *n* is provided for variables for which some patients were missing data.

	Type of Gunshot Wound		
	SI (*n* = 100)	NSI (*n* = 173)	*p*
Location			<0.0001
Hand	85 (85.0%)	57 (33.0%)	
Forearm	6 (6.0%)	38 (22.0%)	
Humerus	2 (2.0%)	33 (19.1%)	
Shoulder	3 (3.0%)	28 (16.2%)	
Elbow	2 (2.0%)	7 (4.1%)	
Clavicle	1 (1.0%)	0 (0.0%)	
Scapula	0 (0.0%)	4 (2.3%)	
Multiple	1 (1.0%)	6 (3.5%)	
Velocity			0.0014
Low	85 (85.0%)	166 (96.0%)	
High	15 (15.0%)	7 (4.0%)	
Type of Gun			0.0036
Handgun	91 (91.0%)	171 (98.8%)	
Shotgun	6 (6.0%)	1 (0.6%)	
Rifle	3 (3.0%)	1 (0.6%)	
Operating Room Intervention			0.42
No	51 (51.0%)	97 (56.1%)	
Yes	49 (49.0%)	76 (43.9%)	
Antibiotics			0.0063
No	5 (5.0%)	28 (16.2%)	
Yes	95 (95.0%)	145 (83.8%)	
Multiple Wounds		*n* = 172	<0.0001
No	92 (92.0%)	114 (66.3%)	
Yes	8 (8.0%)	58 (33.7%)	
Fracture			0.23
No	41 (41.0%)	84 (48.6%)	
Yes	59 (59.0%)	89 (51.4%)	

**Table 3:** Injury Characteristics (*N* = 273). The effective sample size *n* is provided for variables for which some patients were missing data.

	Type of Gunshot Wound		
	SI (*n* = 100)	NSI (*n* = 173)	*p*
Nerve Injury			0.013
No	55 (55.0%)	121 (69.9%)	
Yes	45 (45.0%)	52 (30.1%)	
Nerve Injury Type			0.0035
None	55 (55.0%)	121 (69.9%)	
Axonotmesis	14 (14.0%)	28 (16.2%)	
Neuropraxia	27 (27.0%)	24 (13.9%)	
Neurotmesis	4 (4.0%)	0 (0.0%)	
Nerve Type	*n* = 45	*n* = 62	
Median	3 (3.0%)	11 (17.7%)	0.27
Radial/PIN	3 (3.0%)	12 (19.4%)	0.27
Ulnar	9 (20.0%)	21 (33.9%)	0.55
Digital	30 (66.7%)	10 (16.1%)	<0.0001
LABC	0 (0.0%)	2 (3.2%)	0.53
MABC	0 (0.0%)	1 (1.6%)	0.99
Other	0 (0.0%)	5 (8.1%)	0.16
Vascular Injury			0.013
No	89 (89.0%)	167 (96.5%)	
Yes	11 (11.0%)	6 (3.5%)	
Vascular Type	*n* = 11	*n* = 6	0.017
Brachial	0 (0.0%)	3 (50.0%)	
Radial	1 (9.1%%)	0 (0.0%)	
Ulnar	2 (18.2%)	1 (16.7%)	
Digital	8 (72.7%)	1 (16.7%)	
Other	0 (0.0%)	1 (16.7%)	
Tendon Injury			0.0014
No	75 (75.0%)	155 (89.6%)	
Yes	25 (25.0%)	18 (10.4%)	
Muscle Injury			0.16
No	95 (95.0%)	156 (90.2%)	
Yes	5 (5.0%)	17 (9.8%)	

LABC = Lateral Antebrachial Cutaneous Nerve. MABC = Medial Antebrachial Cutaneous Nerve.

**Table 4:** Wound Classifications (*N* = 273). The effective sample size *n* is provided for variables for which some patients were missing data.

	Type of Gunshot Wound		
	SI (*n* = 100)	NSI (*n* = 173)	*p*
Gustilo–Anderson (GA)			<0.0001
1	16 (16.0%)	69 (39.9%)	
2	67 (67.0%)	93 (53.8%)	
3	17 (17.0%)	11 (6.4%)	
Tscherne	*n* = 98	*n* = 159	0.0048
I	43 (43.9%)	87 (54.7%)	
II	37 (37.8%)	51 (32.1%)	
III	4 (4.1%)	15 (9.4%)	
IV	14 (14.3%)	6 (3.8%)	

## Data Availability

Raw data are not available due to ethical and privacy reasons related to the topic of this investigation (gun shot injury).
